# Cultural and health implications of fish advisories in a Native American community

**DOI:** 10.1186/2192-1709-2-4

**Published:** 2013-03-12

**Authors:** Elizabeth Hoover

**Affiliations:** American Studies and Ethnic Studies, Brown University, Box 1886, Providence RI 02860, USA

**Keywords:** Fish advisories, Native American, Mohawk, Haudenosaunee, PCBs, Superfund, St. Lawrence River, Health risk assessment

## Abstract

**Introduction:**

Fish advisories are issued in an effort to protect human health from exposure to contaminants, but Native American communities may suffer unintended health, social, and cultural consequences as a result of warnings against eating local fish. This paper focuses on the Mohawk community of Akwesasne, which lies downstream from a Superfund site, and explores how fish advisories have impacted fish consumption and health.

**Methods:**

65 Akwesasne community members were interviewed between March 2008 and April 2009. Interviews were semi-structured, lasted from 30–90 minutes and consisted of open-ended questions about the impacts of environmental contamination on the community. Detailed field notes were also maintained during extensive visits between 2007–2011. Interviews were transcribed, and these transcripts as well as the field notes were analyzed in NVivo 8.0. This research received approval from the Akwesasne Task Force on the Environment Research Advisory Committee, as well as the Brown University Institutional Review Board.

**Results:**

Three-quarters of the 50 Akwesasne Mohawks interviewed have ceased or significantly curtailed their local fish consumption due to the issuance of fish advisories or witnessing or hearing about deformities on fish. Many of these respondents have turned to outside sources of fish, from other communities or from grocery stores. This change in fish consumption concerns many residents because cultural and social connections developed around fishing are being lost and because fish has been replaced with high-fat high-carb processed foods, which has led to other health complications. One-quarter of the 50 interviewees still eat local fish, but these are generally middle-aged or older residents; fish consumption no longer occurs in the multi-generational social context it once did.

**Conclusions:**

Human health in Native American communities such as Akwesasne is intimately tied to the health of the environment. Fish advisories should not be used as an institutional control to protect humans from exposure to contaminants; if Akwesasne are to achieve optimal health, the contaminated environment has to be remediated to a level that supports clean, edible fish.

## Introduction

We turn our minds to all the Fish life in the water. They were instructed to cleanse and purify the water. They also give themselves to us as food. We are grateful that we can still find pure water. So, we turn now to the Fish and send our greetings and thanks.

Now our minds are one.

–Haudenosaunee Thanksgiving Address (Patterson 1999)

For traditional Haudenosaunee (also known as Iroquois) people, the *Ohenton Kariwatehkwen* or “Thanksgiving Address” is recited at the opening and closing of important events, as a reminder of essential elements to be collectively thankful for. The translated excerpt above highlights the importance of fish, which historically were an important source of food for many Haudenosaunee nations, including the Mohawk.^a^ In communities such as Akwesasne, the relationship between fish—whose duty it is to cleanse the water and offer themselves as food—and humans—whose role it is to respectfully harvest these fish —has been interrupted by environmental contamination.

Because concepts of health for Mohawk people extend beyond the individual to the community and the environment (Arquette et al. 2002), this interrupted relationship with the fish has resulted in altered diets with resulting health impacts, and the fear that language and culture related to fish will be lost.

Much of the literature on fish advisories has focused on concerns about whether these advisories properly inform minority subsistence fishermen and their families about the risks of consuming contaminated fish (Chess et al. 2005; Beehler et al. 2003; Tan et al. 2011; Imm et al. 2005). Following a conventional human health risk model in which prevention of exposure protects health, the goal of much of this scholarship is ensuring that the fish avoidance message reaches all audiences. Less focus has been given to the impact on communities who follow these advisories, and the feasibility of ever reversing the impact of these advisories even after site cleanup.

Through interviews with Akwesasne Mohawk community members and environmental officials, I explore the impact fish advisories have had on this community, the extent to which the community decreased or ceased their fish consumption, and the unintended health and cultural consequences of fish advisories. I also explore the motivations of people who have decided to continue to eat fish despite the advisories, and conclude with a discussion of future research and outreach needed in the community. The overarching message conveyed by community members and scholars who are pushing for more holistic forms of risk assessment (Arquette et al. 2002; Ranco et al. 2011; Harper et al. 2012; Donatuto and Harper 2008) is that optimum human health cannot be achieved in Native American communities such as Akwesasne until ecological health is achieved as well.

## Background

**Figure 1:**
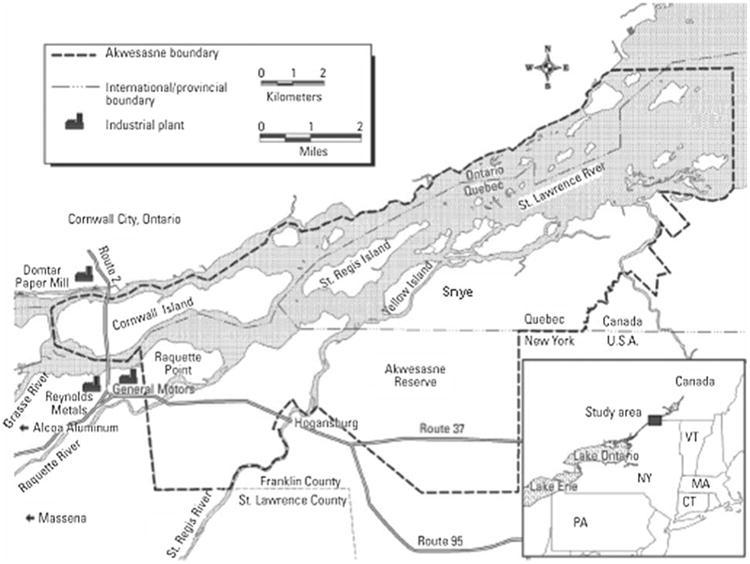
Map of Akwesasne Map available at http://www.ncbi.nlm.nih.gov/pmc/articles/PMC1253751/figure/f1-ehp0113-000272/.

Akwesasne is a Mohawk community of about 13–15,000 people^b^ that shares borders with New York, Ontario, and Quebec. Located at the confluence of four rivers— the St. Regis River, the Raquette River, the Grasse River, and the St. Lawrence River—the community relied for generations on the abundance of fish and wildlife, as well as the rich alluvial soils for farms (see Figure 1).

The St. Lawrence Seaway project, begun in 1954, widened and deepened the river, and created a series of canals and locks that opened the region to ocean-going vessels. In 1957 the Moses-Saunders Power Dam was constructed on the St. Lawrence River and attracted industry with its low-cost hydroelectricity. The Aluminum Company of America (ALCOA) had already established a factory a few miles west of Akwesasne in 1903 on the Grasse River and was joined by the General Motors (GM) aluminum plant in 1958 and Reynolds Metals in 1959, both just west of the reservation, upstream and upwind. On the Canadian side of the river, a paper mill was built in 1881, which was acquired by Dominion Tar & Chemical in 1961 and rebranded as Domtar in 1965.

In 1976 Health and Welfare Canada approached the Mohawk Council of Akwesasne (MCA), the tribal government for the northern half of the community, to determine if there were elevated mercury levels in the people of Akwesasne, since mercury had been identified as a problem in Native communities across Canada. The presence of paper companies such as Domtar adjacent to, and upstream from, Akwesasne led to the concern that mercury could be a threat to this community as well. In 1978 MCA took samples of fish and found PCBs, mercury, and Mirex, which led them to recommend that women of childbearing age, children under 15, and pregnant women cease consumption of fish from the St. Lawrence (Hauptmann 1988:64).

In 1981, two dormant sludge pits filled with polychlorinated biphenyls (PCBs) were discovered behind GM, adjacent to the Raquette Point region of the reservation, and by 1984 the entire 270 acre General Motors site was declared a Superfund site. Following tests done by a NY State wildlife epidemiologist that found high levels of PCBs in fish and aquatic wildlife, an official three-part health risk assessment was developed that examined contaminant levels in fish (Sloan and Jock 1990), wildlife (Skinner 1992) and breast milk (Fitzgerald et al. 1992). Sloan and Jock (1990:26) found that “PCB, dioxin, and mercury throughout the study area exceeded criteria for the protection of piscivorous wildlife.” Tests done on 343 fish collected from 12 locations on the St. Lawrence River near the GM site showed total PCB levels ranging between 3 ppm and 8,000 ppm (lipid basis), which exceeded the tolerance limits established by the federal government of 2 ppm (wet weight) (Kinney et al. 1997:314).

Following tests done around the GM Superfund site, the Health Service for the St. Regis Mohawk Tribe (SRMT), the tribal government for the southern half of the community, issued an advisory in July of 1986 recommending that women of childbearing age, pregnant and nursing women, and children under 15 should entirely avoid the consumption of contaminated fish. They also gave a list of species for everyone to avoid, and a separate list from which residents should only eat one half pound per week.^c^ This news further destroyed the local fishing economy, which had previously supported a number of Mohawk families and had already begun to diminish after the MCA fish advisory.

The results of the breast milk section of the health risk assessment (Fitzgerald et al. 1992), as well as continued studies that resulted from a partnership between the Akwesasne community and the State University of New York (SUNY) at Albany, demonstrated that fish consumption led to higher levels of PCBs in Akwesasne residents. Fingerprinting analyses established that fish caught in the St. Lawrence River offshore from the GM site were contaminated with the same lightly chlorinated PCB congeners as the sediment collected offshore from the GM plant. This contamination was primarily Aroclor 1248, the PCB mixture used in GM's die casting machinery (Hwang et al. 1993; Fitzgerald et al. 1995). This contamination was then traced from fish to humans: breast milk samples from women who ate local fish had a congener pattern that was much closer to perch, the preferred species of fish, than that found in women who ate little or no fish (Fitzgerald et al. 1998; Hwang et al. 2001). Similarly, Mohawk men who ate the most fish had a congener pattern similar to the fish studied (although their serum was more likely to contain more chlorinated PCB mixtures such as Aroclor 1254 and 1260, which have been traced to the Great Lakes, than the local mixture, Aroclor 1248) (Fitzgerald et al. 2007). This series of studies was significant because it demonstrated how PCBs could be fingerprinted as they migrated offsite from an industrial source, and traced into fish and then humans. Many Mohawks I spoke with referred to the St. Lawrence as the “lifeblood” of the community. By the contamination of the river, and hence the fish, Mohawk bodies were subsequently contaminated as well. Therefore cessation of fish consumption, which distanced Mohawks from this river, was lauded by the public health community as a means for preventing further exposure to contamination.

Environmental health studies conducted by the SUNY Albany research team and the Akwesasne community extended into a second Superfund Basic Research Project grant from 1995–2000. Papers published based on this project demonstrated that higher PCB levels in Mohawk participants were potentially connected to abnormal thyroid functioning in adolescents (Schell et al. 2004, 2008, 2009; Schell and Gallo 2010); diabetes (Codru et al. 2007); higher levels of total serum lipids that contribute to heart disease (Goncharov et al. 2008); altered cognitive function in adolescents (Newman et al. 2006, 2009) as well as altered cognitive function in older adults (Haase et al. 2009); earlier onset of menarche in girls (Denham et al. 2005); and reduced testosterone levels in men (Goncharov et al. 2009).

The General Motors Superfund site has been undergoing remediation for decades but may finally be nearing completion. The Environmental Protection Agency (EPA) issued a Record of Decision in 1990 for GM's wastewater lagoons and the contaminated adjacent river sediment and in 1992 for GM's industrial landfill. The dredging of the St. Lawrence River sediments, along with a cap in areas where the cleanup goals were not met, was completed in 1995. From 2000–2004, GM remediated the inactive lagoons at the facility, excavating contaminated sludge and soils, stabilizing them and shipping to an offsite facility. The Raquette River bank soils and sediments were also remediated, and contaminated soils were removed at the toe of the slope of the Industrial Landfill, which reached out to the Raquette Point portion of the reservation. In 2004–2005 GM remediated Turtle Cove, which had been renamed “Contaminant Cove” by NY State Department of Environmental Conservation (NYSDEC) workers. The GM plant closed in May 2009, was bulldozed, and the remains shipped to an offsite landfill in 2011 (USEPA 2011). In 2013–2015 another four lagoons and two sludge pits will be cleaned up, and the edge of the landfill will be pulled back 150 ft from the shore of the St. Lawrence River and reservation boundary. The last of the general site cleanup and restoration activities will occur in 2016 (Arquette 2012). According to the EPA website, current human exposures at this site are under control but contaminated groundwater migration is not (USEPA 2012).

## Methods

In order to explore how a Native American community located downstream from a Superfund site has been impacted by contamination and the ensuing environmental health studies, from March 2008 through April 2009 I interviewed 65 Akwesasne Mohawk community members, ranging in age from 25 to 90, with a majority (*n* = 41, 63%) in their 40s and 50s.^d^ Eight participants were in their 20s to 30s, and 16 participants were older than 60. Those in their 40s and 50s were targeted, as they were the most likely to have taken part in the environmental health studies during the 1980s–1990s. Slightly over half of the interviewees (57%, *n* = 37) were women.

All interviews were semi-structured, lasted from 30–90 min and consisted of open-ended questions about the impacts of the environmental contamination on the community, fish consumption, and current health concerns. During these interviews, I asked 50 interviewees (18 men and 32 women, 68% in their 40s and 50s) directly about their fish consumption, as well as that of their families, and how this may have changed as a result of the fish advisories. It is from this sub-sample that the data for this paper are drawn. Questions included: Do you still eat fish? As much as you used to? If you stopped, or reduced your fish eating, at what point did you do so, and why? Were your changes due to advisories or because you noticed changes in the fish? Do members of your family all eat the same amount of fish?

I made contact with most interviewees through snowball sampling, and in addition approached two members of the SRMT Environment Division, two members of the MCA Department of Environment, and six individuals who were at some time affiliated with the grassroots organization Akwesasne Task Force on the Environment (ATFE). All interviews were recorded and transcribed with all identifying information removed to protect confidentiality if the interviewee chose this option. For those who did not choose to remain confidential, I received permission to use their names. In addition to interviews, detailed field notes were maintained during extensive visits to Akwesasne from 2007 to 2011. Transcripts and notes were then analyzed in NVivo 8.0, a program for qualitative data entry, coding, sorting, and retrieval. Data coding was done in an iterative fashion, with additional readings of the transcripts leading to additional codes as more was learned from the materials. Prior to conducting this research, I received approval from the Akwesasne Task Force on the Environment Research Advisory Committee, as well as the Brown University Institutional Review Board.

## Results and discussion

### Fishing tradition

Prior to the fish advisories issued in the 1980s, fishing had been a central part of the diet, economy, and social culture of Akwesasne for centuries. More than just a means of acquiring a dietary mainstay, fishing was described by community members as a livelihood, a lifestyle, and a culture. Almost everyone I spoke to in the community had a connection in some way to fish or fishing. The process of catching and cooking fish out of the river was at the root of many of the interviewees' childhoods and something that connected them to their ancestors. People in their 50s on up through their 90s recalled with youthful excitement their childhood experiences of going to the fish boxes that each family kept on the shore of the river to pull out the evening's supper. People fondly reminisced about fishing with their fathers on the river, helping their fathers prepare fishing equipment, or helping their mothers clean and cook the fish. Species such as sturgeon, perch, and bullhead were mentioned most frequently, and they were eaten smoked or fried. Fish was eaten several times a week for an ordinary dinner, and in large quantities at “fish fries” to celebrate special occasions and family gatherings.

When fish advisories were issued that called for people to diminish or eliminate fish from their diet, many residents felt that they had lost more than just omega-3 fatty acids from their diet; another part of their culture was being eroded by outside influences. A cessation in fishing gradually diminished Mohawk culture in several ways. As Henry Lickers describes, the language and culture around tying knots in nets as well as the social interactions that occurred around the process of creating these nets are lost when there is no longer a use for those nets:

People forget, in their own culture, what you call the knot that you tie in a net. And so, a whole section of your language and culture is lost because no one is tying those nets anymore. The interrelation between men and women, when they tied nets, the relationship between adults or elders and young people, as they tied nets together, the stories… that whole social infrastructure that was around the fabrication of that net disappeared (interview 10).

Similarly, the language around the names and descriptions of certain fish is lost. As one older man described to me, “A lot of that has been forgotten, the fish names in our language. Because a lot of the fishermen when they go fishing they talk about their Indian names to them, there is no English part of it, but that has been sort of forgotten now” (interview 39). One young mother, Randi, described how even though the youth were learning the Mohawk language, words related to activities no longer widely practiced, like fishing, “are never going to be spoke again because those things are now in the past… so only very elderly people are going to know those words, so that's a loss” (interview 54).

### Changes in community fish consumption

Data gathered during the health studies demonstrated a decrease in fish consumption among pregnant women (Fitzgerald et al. 1995, 2004) and Mohawk men (Fitzgerald et al. 1999). These papers cite fish advisories as the reason for the decline in fish consumption. Many of the Akwesasne community members I interviewed also described a decline in fish consumption, attributed to the fish advisories, but also to visual changes in the fish and a diminished fish population. On the other hand, some have maintained fish consumption based on a cultural connection that ties them to fish or because they felt their age precluded them from the more pressing reproductive and developmental concerns associated with the main target demographic of the fish advisories.

When asked whether they still ate local fish, three-quarters of the interviewees I spoke with (37 out of 50) replied that they had either dramatically decreased or entirely ceased their local fish consumption, even though for most of them it was previously an important part of their diet. Of those who had decreased fish consumption, eight interviewees described fish meals as a rare, special occasion treat. As Howard, an elder in his 80s explained, “I eat fish once or twice a year, not like everyday” (interview 13). Another woman remarked that when she does have a little bit of fish on these occasions, she worries, “You know in the back of your mind that you're going to be glowing (laughs). You know what I mean? You know it is there, the fear is still there” (interview 27). Others have sworn off fish entirely and express aversion even to the idea. As Gina described, the only circumstance under which she would eat local fish would be, “Maybe if somebody raised them and then threw them in the river and you caught them that same day” (interview 4). Even though the fish advisories targeted mainly women of childbearing age and children, three of the men I spoke with shared what they saw as a common sentiment among men: if it was bad for the women, they should not eat it either. Nineteen (19) of the interviewees specified that not only had they given up eating fish, but so had the rest of their families.

Even prior to the announcement of fish advisories, which 29 interviewees indicated to me was what drove them from eating fish, some residents began noticing or hearing about visual clues that fish were not safe. One Raquette Point resident, Mark, described to me catching “fish with humongous tumors on them,” or “funny color eyes” (interview 34). Others (*n* = 6) recalled how the fishermen were catching fish with black spots on them, with bugs inside, or with sores. Another man, Robert (interview 32), remembered cutting open fish with black spines, which he described as resulting from heavy metals contamination. Gina recollected, “We'd see like a big black spot on them or a glob of green and they would tell us that's what the PCBs are doing” (interview 4). Whether or not all of these physical changes witnessed in the fish can be attributed to PCBs is debatable, but more importantly, seeing or hearing about these physical deformities discouraged Mohawks from eating local fish.

Problematically, not all contaminated fish showed such visual cues. In 1985, NY State wildlife epidemiologist Ward Stone took samples of a sturgeon caught by Mohawk fishermen in order to test it for PCBs. When he came back with his results, which showed the fish to contain levels of PCBs above what the USDA considered safe for consumption, he was aghast to learn that the fishermen had already eaten the sturgeon.

The fact that fish could be contaminated without showing visible evidence led many community members for whom it was financially feasible (including 11 of these interviewees) to rely solely on outside sources of fish. For three related interviewees, this fish was coming from as far away as British Columbia. For three others it was from other communities nearby, such as Tyendinaga.^e^ Robert explained how he gets his perch “out of upper Canada where they're not polluted yet” (interview 32). Some realized the irony in that they were probably still consuming contamination, just from another place they were less familiar with. Chris, an iron worker who had spent most of his life in Akwesasne but had also traveled extensively, exclaimed how “people think that if the fish comes from someplace else rather than right here, then it is ok. They don't realize every Great Lake dumps into the next Great Lake, which dumps into the St. Lawrence River. It is one big sewer” (interview 29). To avoid even the concern, four interviewees reported that they would only eat fish from the supermarket. As Alice, a lab technician for the health clinic described, “So we end up being supermarket Indians, buying tilapia from Hannaford's, not so much perch or walleye” (interview 62). For those who can afford to buy fish from outside the community this helped to satisfy the nutritional void created by the fish advisories, but not some of the social and cultural roles described earlier.

Henry Lickers, who works for the MCA Department of Environment, described the rapid decline in fish consumption since moving to the community 30 years ago. When he first arrived in the community, he reports that 90% of the people he visited were eating fish. Then as the fish advisories became more prominent, people began changing their behavior. When he would stop by their house at dinnertime:

Suddenly, the old man or whoever it is was cooking the fish would put it in the cupboard and shut the door. And then they would be cooking something else, you know. “Well, you know, Henry has been talking about this. And you don't want to show him that you don't believe what he's talking about, because I really like fish, you know. And besides, I'm over 60, and it's not going to hurt me. And I don't want to have any more kids, so I'm okay.” But you got funny things like that occurring (interview 10).

Henry no longer eats fish because he feels that he has a responsibility to set an example: “I don't eat fish from the St. Lawrence. I believe the same way, if people saw me eating, then they would say ‘oh, then we can go back.’ And I don't think that that's responsible. If I'm going to tell them not to, well then I better not as well.” At the same time, he recognized that some people do still eat fish, even if they do not openly admit it. It is possible that for the earlier cited studies in which Fitzgerald was documenting a decline in fish consumption, as well as in the interviews I was conducting, that people under-reported their fish consumption because they recognize that according to the fish advisories they are not supposed to be eating fish, and they do not want to be judged for their choices.

One-quarter of the interviewees (13 out of 50) I asked about fish consumption expressed that they continued to eat local fish because they felt a cultural obligation to do so (*n* = 4), were not concerned about the warnings based on personal experiences (*n* = 4), or have decided to resume fish consumption now that they are no longer in their child-bearing years (*n* = 5). Richard described to me the traditional connection and responsibility that Mohawks and the fish have to each other, and for this reason, he continues to eat the fish. As he described, the Creator put the food in the water:

We give thanks for that food and we have to use it… I mean it doesn't make sense scientifically, but it makes sense spiritually and mentally that you should eat that, you know. You can't just put it aside and say, “well your work is not good enough,” or something, you know? They're still given out what their original instructions were, and it's us that are at fault, it's our fault that they're like that, you know (interview 20).

Even though as a Mohawk he is not responsible for the contamination that has affected the fish, as a human being he is implicated in the problem, and therefore it is even more important that he works to maintain this relationship with the fish. Because the job given by the Creator to fish is to offer themselves as food, and the job given to humans is to respectfully harvest these fish, people like Richard who are working to maintain tradition feel obligated to maintain these roles. I heard similar narratives around the preservation of heritage seed varieties: the duty of these seeds is to sprout every spring, and the duty of humans is to plant them. If Mohawks fail to plant these seeds, “the plants will go back to the Sky World because they volunteered to come to the earth to help man to survive” (Brenda, interview 21). The concerns around the disuse of fish and heritage seed varieties stem back to cultural stories about ungrateful humans who have their food sources taken from them and who only regain them after learning lessons about maintaining ceremonies and traditional roles.

For some community members, an impression that the site remediation has led to lower levels of contamination in the fish or a nuanced understanding of more and less contaminated species and methods of preparation has contributed to their choice to eat local fish. One woman, who worked on the environmental health studies as a field assistant and was part of ATFE, described how her family continued to eat fish, and her kids “love it. They eat it whenever they can get it. I know that the area has been remediated and the fish isn't that bad anymore. So I hadn't told them not to eat it. So we just continue” (interview 26). Another ATFE member, Joyce, described how “the levels with the fish going down, the PCB levels going down… I feel more comfortable eating fish now. So I don't think I'm going to pick up that much contamination with PCBs anymore” (interview 16). Another woman, Randi, who had relatives who worked on the health studies and with ATFE, noted that many people in the community have vilified the consumption of all fish. She noted that if caught eating local fish, pregnant women could expect a similar reaction as though they were smoking or drinking. She expressed disappointment that the only lesson that people seemed to take away from the health studies is not to eat fish:

I feel like sometimes I could try to educate people about what fish is good for you and what is bad for you, but sometimes it is just, why bother? You know my 30-second conversation is not going to undo 12 years of ingrained messages—“don't eat any more fish”… So I don't fight it too much, I just eat my fish in private (interview 54).

She laughed that people could eat fast food and then criticize her for eating fish. Similarly, an individual employed by the Environment Division stated that he felt the nutritional benefits of fish outweighed the potential contamination in fish since the beginning of the remediation of the industrial sites.

For other community members, personal and family experience with fish that have not led to ill health has encouraged them to continue to eat local fish. Agnes, who also worked on the environmental health studies, described how “we were brought up by the river and on the river. We were brought up to fish, we were brought up to swim in the river, and we were brought up on a boat. I don't have no fear of contamination. It was just a part of my life” (interview 59). She still eats fish as well. Nelson, a farmer and construction worker from the Snye region of the reservation, expressed skepticism towards all of the fish advisories because “we've been fishing all of our lives and we're still here. And my aunt, we just buried her last week. My great aunt was 102” (interview 40).

To a great extent, fish consumption in the community is divided along generational lines. In several families, members described to me that younger women who were planning to have children would not eat fish, but older women would go back to eating fish. For example, as Brenda, who is in her 50s, expressed, “I'm not young anymore so it doesn't matter. I eat the fish” (interview 21). Elizabeth, who is also in her 50s, also described how she went ten years without eating fish and would not let her kids have any. But she has gone back to it recently: “Let's just say I'm getting older now. I don't care, I love fish” (interview 22). However, even though this more advanced generation has returned to fish consumption, many interviewees were not convinced that the younger generation, even if given the chance, would eat fish. Middle-aged and older community members who had gone back to eating fish noted that they had not raised their kids to do so because of the warnings, and now they “didn't develop a taste for it” as Joyce describes. Seven interviewees described how their children and grandchildren currently had no desire to eat fish and are unlikely to show an interest in it even if it were determined to be clean at some point. As Agnes described, “They weren't brought up with the fish so they're not going to turn around and change their ways” (interview 59).

Even aside from the issue of contamination, ten of the community members I spoke with expressed the opinion that the fish population is too low to support the community. As Joyce, whose father was a fisherman, noted, “There isn't enough of a fish population to make a living off of anymore.” She and others referred to the cormorant, a voracious bird that is new to the area and has been decimating fish stocks, especially the perch populations. Others pointed to the dams and locks now in place on the St. Lawrence River that prevent the fish from getting upstream and spawning as they once did. Ernie, a 90-year-old elder who witnessed the transformation of the St. Lawrence from a river to a seaway, described how, in the process of dredging channels and blasting through rock ledges in the river, the fish spawning grounds were destroyed.

[The blasting of rocks] affected the spawning grounds of fish, not only by the blasting but also because of when the blasting was done, they had to clean out all the broken bottom soil and then deposit it somewhere. And of course the easiest place to do it were the inlets and the bays where there were spawning areas and so for a long time fish couldn't make a living out there and so a lot of their work was not done. The fish as you know, have sort of a cleaning action there in swimming – absorbing the water and taking in contaminants, deposit it down in the bottom of the river, so getting it out of the way. And so now we had to do without the fish for years.

As mentioned in the Thanksgiving Address, part of the instruction given to fish is to cleanse and purify the water. As Ernie has noted, this job has been hampered by changes made to the river. As Richard remarked above, the other instruction given to the fish is to offer themselves as food. Contamination released into the river and some residents' concerns about taking this contamination into their own bodies have dramatically altered the feasibility for this job to be carried out as well.

### Costs and benefits of fish consumption

For some, current rates of diabetes, heart disease, and obesity among Mohawks have led them to wonder if trading local fish for inexpensive processed foods was the healthiest decision. Currently about 25% of the community suffers from diabetes (Rourke 2009, director of “Let's Get Healthy” program, personal communication). Of the 38 interviewees who expressed opinions about the causes of diabetes in the community, half of them (*n* = 19) pointed to a change in diet brought about as a result of the contamination of the environment. One man, Richard D., who works for the MCA Department of Environment, wants someone to do a study to see who currently has better health: the individuals who ignored the fish warnings and continued to eat a traditional diet or those who heeded the warnings to avoid fish and instead substituted a high carbohydrate, high fat diet, “like me, and developed obesity and blood pressure and diabetes and stuff like that. Like who's better off? I'd like to see a study of that” (interview 45). He attributes the high rates of diabetes to the community's collectively changed diet. “There's a lack of healthy food here. I mean, used to be we'd get bullheads out of here for consumption. I wouldn't touch them now.” So while the fish advisories were intended to protect Mohawk health, the shift in diet away from fish to other affordable sources of nutrition also caused health problems.

Jim, who worked for the SRMT Environment Division at the time the contamination was discovered, expressed mixed feelings for the fish advisories that strongly encouraged people to change their diets: “The problem we didn't anticipate though was the change in the diet and the change in lifestyle we feel has contributed to the diabetes in the community and to the other illnesses in the community that has occurred since then. So that concerns me” (interview 36). In criticizing the conventional risk assessment model, Akwesasne community members Tarbell and Arquette (2000) noted that sometimes the greatest health effects are seen outside of chemical exposures and are thus not included in risk assessments. At Akwesasne, health was impacted by the environmental contamination even without the ingestion of fish: fear of exposure led to the replacement of this low-fat source of protein and other important nutrients with high-fat and high-carbohydrate sources of food. Tarbell and Arquette (2000:102) posit, “Diabetes is on the rise because more people no longer eat traditional foods and no longer participate in cultural activities that once provided healthy forms of exercise.” SUNY Albany researchers conducting environmental health studies with Mohawk adolescents also noted that while the community might have decreased their exposure to fish-borne contamination, they have lost a primary source of protein and other important nutrients such as calcium, iron, zinc, and omega-3 fatty acids (Fitzgerald et al. 2004). The replacement of fish with cheap foods has had the effect of “further exacerbating chronic, diet-related health problems in the community, such as diabetes and cardiovascular disease” (Schell et al. 2003:961).

Despite these concerns, these researchers have expressed that reducing fish consumption has been more beneficial to health than continued fish consumption because of the risks of contaminant exposure. In a recent paper, Schell et al. (2012) assessed the benefits of consuming less local fish (lower PCB levels in the youth they tested) versus the costs (cultural loss and higher rates of diabetes and obesity). They concluded that the more holistic risk-based environmental decision-making proposed by ATFE members Arquette et al. (2002) should be better considered by regulatory agencies, and human biologists should give more focus to the non-nutritional components of many foods, such as persistent organic pollutants (POPs). David Carpenter (personal communication, 2008) has argued that, especially since some studies done at Akwesasne have connected PCB levels with potential health effects, the cessation of fish consumption was the best option for Mohawks, although more should have been done to help people find healthier food substitutes. Carpenter also questioned whether the touted benefits of fish consumption are enough to counterbalance the potential impacts of the contaminants (Bushkin-Bedient and Carpenter 2010). Turyk et al. (2012) argued that most of our knowledge about the nutritional benefits of fish consumption is based on marine fish, which generally have higher concentrations of omega-3 fatty acids than freshwater fish. They pointed to studies by Philibert et al. (2006) and Godin et al. (2003) that found no association between local fish intake and serum omega-3 fatty acids in Great Lakes fishermen. Since omega-3 fatty acids are one of the most highly cited health-promoting compounds in fish, this has led Turyk et al. (2012) to conclude that we do not have enough data to quantitatively analyze the costs and benefits of the consumption of fish from the Great Lakes and the St. Lawrence River.

At a recent community meeting (1/11/12) questions arose around whether the fish advisory had been the best course of action, considering the unintended health consequences such as obesity and diabetes that have been linked to a more modern diet. The researchers hosting the meeting acknowledged that their data demonstrated that youth born before the fish advisory (1985) had higher PCB levels than those born after, demonstrating the effectiveness of the advisory in lowering PCB body burdens (see Schell et al. 2003, 2012; Gallo et al. 2011). One participant pushed further—what about now, after the remediation work that has been done? In the discussion that followed, the general consensus was that more testing needed to be done on local fish populations to determine current contaminant levels. An Environment Division employee present at the meeting announced that there were plans in the coming year to collaborate with the NYSDEC to repeat a fish study done in 1988, and efforts were being made to convince EPA to do fish monitoring at the remedial sites. When I interviewed an Environment Division employee and asked if the fish will ever be considered safe to eat again, he replied, “I think so. I think that'll be as clean as the fish upstream.” He remarked that the farther you go from GM, the cleaner the river gets, and the cleaner the fish get.

So, when you go down into Snye (a region of Akwesasne east of GM), we've taken the samples of fish and the fish are pretty clean. I think they are – we are still being cautious; we still don't want to say yes, you can eat the fish, because we are still not sure what the proper level is that's safe. Is it going to be 0.05 parts per million, is it going to be 0.5 parts per million you know, 0.005… you know there is still science that's going on. So, we want to be very sure of what we're doing before we say it's okay. I think it's going to happen eventually you know” (interview 15).

In order for the tribal government to be able to properly advise residents about the risks and benefits of eating local fish, more testing needs to be done, and the results conveyed in a manner that the community can understand and apply.

### Risk avoidance and reduction

The case of Akwesasne is illustrative of unintended health and cultural consequences of relying on risk avoidance rather than risk reduction methods of preventing human exposure to contamination (O'Neill 2003 NEJAC 2002). *Risk reduction strategies* look to risk-producers to prevent or eliminate environmental contamination in order to avoid human exposure. In the case of Akwesasne, risk reduction strategies would have entailed greater monitoring of General Motor's operations to prevent decades' worth of PCB contamination to the surrounding area. A greater enforcement of risk reduction would also have led to the immediate and complete removal of PCB-contaminated waste from the GM site, rather than a decades-long cleanup that resulted in a 12 acre landfill, and a reliance on continued fish advisories to prevent Mohawks from being exposed to contamination. *Risk avoidance strategies* call upon the risk-bearers to alter their practices so as to avoid the harms of exposure to contamination. Fish advisories are an example of this strategy: the onus is on the risk-bearers, in this case the fish consumers, rather than those who caused the risk.

O'Neill (2003) points out that reducing human health risks by targeting the first link in the chain that connects environmental contamination to human health—in this case removing the source of the PCB contamination— means that ecological health benefits as well. Intervening late in the chain and breaking the link at the point of human exposure, in this case by preventing fish consumption, leaves a greater amount of contamination unabated, has greater negative environmental effects, and is a form of cultural discrimination.

Risk-avoidance strategies also rely implicitly on the assumption that there are readily available substitutes for local fish and the customs that accompany their gathering (NEJAC 2002). As we have seen from the case in Akwesasne, these substitutes were not available to all residents, and there are no substitutes for the cultural activities and knowledge exchange that once happened around fishing. Fish was not just a source of protein for Mohawks, but a cultural object of importance that could not easily be factored into the assessment of mortality and morbidity risks that currently comprise health risk assessments (Donatuto et al. 2011). In order to properly calculate whether risk-reduction strategies are an acceptable solution to environmental contamination, it is necessary to look beyond the risk to a population of cancer deaths, and consider the threats to a healthy, culturally specific lifestyle as defined by the Mohawks themselves. This more complete assessment would include cultural indicators such as access to a traditional diet and the passing down of traditional knowledge (Johnson and Ranco 2011).

While fish advisories were necessary in the 1980s to protect Mohawk health when GM's contamination was first discovered, EPA has not made any concerted attempts to ensure that advisories will only be necessary in the short term (O'Neill 2003). While fish advisories are often necessary to protect human health in the short term, there is a need to emphasize more permanent and judicious fixes to problems of contamination. The EPA's most recent Five Year Report about the GM site states that “remedial actions have been completed in the St. Lawrence River, Raquette River, and Turtle Cove, and, *when combined with the existing fish advisories*, these measures address unacceptable exposure pathways in these areas” (USEPA 2010: ES1; *italics* mine). This report takes for granted that fish advisories are an acceptable tool for preventing human exposure to contaminants, similar to a cap on the river bottom that isolates contaminated sediment. Acceptance of the ongoing fish advisories allows GM and the EPA to avoid a more thorough and permanent cleanup.

## Conclusion

Human health in Native American communities is intimately tied to the health of the environment. Akwesasne Mohawks (Arquette et al. 2002:262) have defined health as “based on peaceful, sustainable relationships with other peoples including family, community, Nation, the natural world, and spiritual beings. Health is supported by the solid foundation of a healthy natural world.” Human health in a community such as Akwesasne cannot be understood independent of the health of the natural environment, and especially in this case, the health of the fish.

When Mohawk mothers who participated in the breast milk study demonstrated that removing local fish from their diets decreased the PCB contamination in their breast milk, they showed that exposure to PCBs was avoidable, and thus according to conventional risk assessment the problem was eliminated. What was not predicted, and the reason why indigenous scholars such as Arquette et al. (2002) and Ranco et al. (2011) are calling for more holistic risk assessment, was that avoiding fish consumption had other health implications, both physical and cultural. For community members who continue to consume fish, this consumption is often socially fractured rather than part of a broader social context. As described above, fish is consumed along generational divides by Mohawks who see themselves as beyond their childbearing years, or “in private” as one woman described, in order to avoid judgment.

If the Akwesasne community is going to achieve optimal health, the contaminated environment has to be remediated to a level that supports clean, edible fish. This will not happen as long as fish advisories are considered an acceptable institutional control to protect humans from exposure to contaminants. The accepted culture around environmental cleanup must shift away from one of risk avoidance, a distributive justice situation where those who did not benefit from industry are forced to pay the price in order to protect their health. At the General Motors Superfund site in Massena, economic considerations were given priority over the health and culture of Mohawk people, in an attempt to secure a source of employment for the residents of Massena. Despite these efforts, and $49.5 billion in bailout money, General Motors filed for bankruptcy in June 2009 and spun off a holding company to handle its idle properties, Motors Liquidation Corp. When it emerged from bankruptcy, GM was freed of responsibility for rehabilitating dozens of toxic waste sites in 13 states where it had manufacturing plants. Revitalizing Auto Communities Environmental Response, or the RACER Trust, is an environmental trust that assumed ownership of 89 former General Motors properties and is tasked with completing remediation and selling off dozens of former GM properties, including the one adjacent to Akwesasne. The new General Motors, freed of its old liabilities, is struggling back towards success in the auto market, while the Massena town residents who fought to shelter the company from high cleanup expenses are left without jobs, and the Mohawk are left living adjacent to an industrial landfill that they suspect will continue to pollute their environment.

Researchers and environmental officials working in Akwesasne have stated that a new round of studies determining current contamination levels in the fish is necessary to determine the safety of local fish consumption. Researchers have additionally called for studies of the nutritional content of the fish in order to properly conduct a cost-benefit analysis as to whether the nutritional benefits of the fish outweigh the risks posed by contamination.

Once information about local fish is obtained, it could be disseminated via a model illustrated by DeWeese et al. (2009), who produced advisory maps for Anishnaabe communities that provided lake-specific, risk-based, culturally sensitive consumption advice on color-coded maps for two groups: children under age 15 years and females of childbearing age, and males 15 years and older and females beyond childbearing age. Because the maps were easy to read and developed in conjunction with community members, DeWeese et al. (2009) found they significantly increased the percentage of survey participants who indicated awareness of advisory information and increased preference for smaller walleye, which contain lower levels of contamination than larger fish. After future testing of Akwesasne's local fish population, similar maps could be produced and distributed as a way of making this information accessible to the community. At Brown University,^f^ we are currently developing a project with the Narragansett Tribe in which local fish will be tested, and based on the results, cards will be developed and distributed to fishermen to keep in their tackle boxes to help them determine which species of fish to keep and which to toss back based on contaminant levels. We are also developing fish puzzles for families that help illustrate which parts of the fish are more or less contaminated in order to facilitate the consumption of the less contaminated portions. Each of these small projects described above can help a community to maintain some level of fish consumption rather than cutting it entirely from their diet, as most people at Akwesasne have even though environmental specialists in the community point to some alternatives. What should be highlighted though is that these alternate species and methods of preparation should not be considered as a solution, but rather a step along the process towards remediating environmental contamination to a level in which culturally preferred fish species and methods of preparation can be safely utilized again.

Akwesasne is a case where measures taken to protect community health—adherence to fish advisories to prevent exposure to contamination—have inadvertently led to diet-related health consequences as well as concerns around the loss of language, culture, and social connections attached to fishing. While Akwesasne community members, environmental specialists, and their allies in the scientific community are continuing to push for additional cleanup and fish sampling, this case should also serve as an example of why risk avoidance methods such as fish advisories should not be considered a viable long-term solution to preventing human exposure to contamination.

## Footnotes

a The Haudenosaunee, also known as the Iroquois or Six Nations, are a confederacy of nations traditionally from the region that is now New York State, but now spread across New York, eastern Canada, Wisconsin, and Oklahoma. The six nations of the confederacy include the Mohawk, Oneida, Onondaga, Cayuga, Tuscarora, and Seneca. Akwesasne is one of five Mohawk reservations, which also includes Kahnawake and Kanesatake in Quebec and Wahta and Tyendinaga in Ontario. The Six Nations reservation in Ontario is home to Mohawk people as well as Haudenosaunee people of the other five nations as well.

b An estimate of Akwesasne's population is given because residents in this community are notoriously difficult to enumerate. Akwesasro:non have been suspicious of, and resistant to, national censuses, and some residents are enrolled with both the St. Regis Mohawk Tribe and the Mohawk Council of Akwesasne. This estimate was used in establishing the demographics of the community for the extensive health studies conducted by SUNY Albany (see for example Fitzgerald et al. 1998 and Schell et al. 2009).

c Lactating women and women of childbearing age are to avoid all contaminated fish (carp, catfish and suckers). Adults should only eat one half pound per week of yellow perch, pumpkinseed, rock bass, whitefish, bowfin. Everyone should avoid carp, channel catfish, walleye, pike, red horse suckers, white suckers, and brown bullhead.

d Participants were not asked their exact age. Some participants offered their precise age, others indicated a general age range (e.g., 50s) during the interview.

e A Mohawk community about 150 miles west of Akwesasne in Deseronto, Ontario.

f The Community Engagement Core of the Brown Superfund Research Project, consisting of the author, Marcella Thompson, and Robert Vanderslice, working in conjunction with Dinalyn Spears, head of the Natural Resources Department of the Narragansett Indian Tribe.
